# Selenium‐sensitive *miRNA‐181a‐5p* targeting *SBP2* regulates selenoproteins expression in cartilage

**DOI:** 10.1111/jcmm.13858

**Published:** 2018-09-24

**Authors:** Zixin Min, Yuanxu Guo, Mengyao Sun, Safdar Hussain, Yitong Zhao, Dongxian Guo, Huang Huang, Lisong Heng, Fujun Zhang, Qilan Ning, Yan Han, Peng Xu, Nannan Zhong, Jian Sun, Shemin Lu

**Affiliations:** ^1^ Department of Biochemistry and Molecular Biology School of Basic Medical Sciences Xi'an Jiaotong University Health Science Center Xi'an Shaanxi China; ^2^ Department of Orthopedics and Traumatology Honghui Hospital Xi'an Jiaotong University Health Science Center Xi'an Shaanxi China; ^3^ Key Laboratory of Environment and Genes Related to Diseases Ministry of Education Xi'an Shaanxi China

**Keywords:** cartilage, *miRNA‐181a‐5p*, SBP2, selenoprotein

## Abstract

Selenium (Se) deficiency brings about defects in the biosynthesis of several selenoproteins and has been associated with aberrant chondrogenesis. Selenocysteine (Sec) Insertion Sequence (SECIS) and SECIS binding protein 2 (SBP2) interaction is a very critical node for the metabolic balance between Se and selenoproteins. The *Gpx1*,* Gpx4* and *SelS* have different binding affinities with SBP2 in cells. According to our results, both *miR‐181a‐5p* and SBP2 appeared to be selenium‐sensitive and regulated the expression of selenoproteins in C28/I2 cells under Se sufficient environment. However, they showed significantly opposite expression trend in Se deficiency rats cartilage and SeD C28/I2 cells. The *SBP2* is a direct target gene of *miR‐181a‐5p* in C28/I2 cells as determined by reporter gene and off‐target experiments. And the *miR‐181a‐5p* could regulate SBP2 and the selenoproteins in C28/I2 cells. Depending upon the Se supply levels, C28/I2 cells were divided into three groups, that is normal Se, SeD and SeS, which underwent through a 7‐day Se deprivation process, then *SBP2* was knocked‐down and overexpressed in all the groups. Moreover, the selected selenoproteins were down‐regulated in second‐generation low Se diet rat cartilage. The selenoproteins expression was decreased by Se deficiency which depended on the Selenium‐sensitive *miR‐181a‐5p* to participate and regulate SBP2 at post‐transcriptional level. It involves a series of antioxidant and ECM (extracellular matrix) genes, to overcome the ROS‐related stress for the protection of essential physiological functions and to maintain the balance between anabolism and catabolism of the cartilage.

## 1 INTRODUCTION

Selenium (Se) is one of essential biological trace element in mammals and is incorporated into selenocysteine (Sec) that is 21st proteinogenic amino acid. Sec is an active‐site residue essential for catalytic activity of selenoproteins (Sel), named after the Se.^1^, ^2^, ^3^ Sel are involved in various biological processes, such as antioxidative stress, anti‐tumour and especially the development.^3^, ^4^ Sec is encoded into the Sel by the genetic code “UGA” that is commonly a termination codon in cells.^5^ Sel biosynthesis is regulated by several special cis‐trans elements and trans‐acting factors, such as selenocysteine insertion sequence (SECIS) and SECIS binding protein 2 (SECISBP2 or SBP2).^6^, ^7^ SECIS is located at 3′‐untranslated region (3′‐UTR) of Sel mRNA and can bind with SBP2. SBP2 functions to carry Sec‐tRNA^Sec^ into ribosomal “A site” that recognizes the “UGA” as the codon for Sec during Sel synthesis.^6^, ^7^

Se deficiency leads to altered synthesis of several selenoproteins, affecting chondrogenesis.^8^ This results in epiphyseal plate abnormalities and articular cartilage degradation.^9^ Representatively, Kashin‐Beck disease (KBD), an endemic osteoarthropathy in Se deficient regions of China, is characterized by pathological chondronecrosis in the deep zones of articular cartilage and growth plates from different peripheral joints of the endemic children.^10^, ^11^, ^12^ Further, Se supplementation is able to protect the Se deficient rats from epiphysial growth plate abnormality and has been used in the prophylactics of KBD by unknown mechanisms.^9^, ^13^, ^14^

Intriguingly, osteo‐chondroprogenitor‐specific deletion of the selenocysteinyl tRNA^Sec^ gene leads to the phenotypes similar to KBD, particularly showing chondronecrosis and abnormal skeletal development in mice.^15^ It is revealed that the termination codon “UGA” is subjected to in‐sufficient Sec‐tRNA^Sec^, because of which the inactive truncated selenoproteins are produced.^16^ Parallelly, the short inactive fragment of TrxR1 with a two‐amino acid‐truncated C‐terminal motif could make human lung carcinoma A549 cells die.^17^ However, it is poorly understood how Se regulates the selenoprotein biosynthesis in cartilage. Particularly, to find the first‐hand factor of Se‐sensitivity is of great importance to understand the mechanism. In addition, there is little known about the regulatory pathway mediated by SBP2 from Se deficiency to selenoprotein biosynthesis in cartilage.

On the other hand, it has been observed that *Dicer*‐deficient mice suffer from a severe deficiency in bone development with proliferating chondrocytes and expansion of the hypertrophic region in the limb bud,^18^, ^19^ suggesting that miRNAs regulate both proliferation and differentiation of growth plate chondrocytes. MiRNAs have been demonstrated to be associated with both cartilage homoeostasis and development, especially the cartilage‐specific miR‐140.^20^, ^21^ About 30 miRNAs are differently expressed during the development of rats femoral articular cartilage.^22^ For instance, miR‐337 could influence cartilage‐specific gene expression in chondrocytes.^23^ It would be very exhilarating if we could find a “selenium‐sensitive miRNA” to regulate the expression of SBP2 in cartilage.

In this study, *miR‐181a‐5p*, one of the differently expressed miRNAs during cartilage development,^22^ was predicted as a target of *hSBP2*with TargetScanHuman7.1. Coincidentally, it could repress the expression of Cyclin A2(CCAN2) and Aggrecan(ACAN) in chicken chondrocytes that may act as a negative feedback for cartilage homoeostasis.^24^ However, to understand the physiological roles of *miR‐181a‐5p* in cartilage, further investigation was required. *GPx1*,* GPX4* and *SELS* were selected as selenium phenotype markers in cartilage or chondrocytes, as they not only are regulated in cartilage by Se deficiency and Se supply,^9^, ^25^ but also are the representatives of different subcellular localizations: cytoplasm, mitochondria and endoplasmic reticulum, respectively. Hence, the detailed regulatory relationship among “low‐selenium, miRNA, SBP2 and selenoproteins in cartilage” was investigated during this study.

## 2 MATERIALS AND METHODS

### 2.1 Rats

The inbred Dark Agouti (DA) rats^9^, ^22^, ^23^ were raised in a climate controlled environment, housed in polystyrene cages containing wood shavings and were fed standard rodent chow and water ad libitum*,* in the SPF animal house of the Department of Biochemistry and Molecular Biology, Xi'an Jiaotong University, Health Science Center. The DA rats originated from the Section of Medical Inflammation Research, Lund University, Sweden. The two generation low‐selenium rat model was established as described previously, including Se deficient diet group (SeD) and Se sufficient diet group (SeS).^9^ The experimenters were blinded to the Se condition while processing data and making exclusion decisions. All procedures were in accordance with the Animals (Scientific Procedures) Act, 1986 (UK) (amended 2013). All sections of this report adhere to the ARRIVE Guidelines for reporting animal research.^26^ A completed ARRIVE guidelines checklist is included in Checklist S.

### 2.2 Cell culture

C28/I2 cell line, human juvenile chondrocytes, were maintained in Dulbecco's modified Eagle's medium/F‐12 medium (DMEM/F12, Hyclone, USA) containing 10% foetal bovine serum (FBS, Gibico, USA) in a humidified incubator with 5% CO_2_ at 37°C. For Se deficient and sufficient experiments, C28/I2 cells were preconditioned with medium containing 1% FBS at least for 7 days resulting in Se depletion (Se deficient condition, SeD). Then, 50 nmol L^−1^ sodium selenite (Se, Sigma) supplementation was performed to rescue the Se level (Se sufficient condition, SeS).

### 2.3 Histological staining

Knee joints fixed with 4% paraformaldehyde (PFA) were tenderly decalcified in 10% EDTA liquid for 4 weeks. Subsequently paraffin‐embedded, dissected into 5‐μm‐thick pieces and stained to observe the morphological changes in the epiphyseal plate. All the sections were then stained with conventional haematoxylin and eosin (H&E), safranin O and fast green dyes.

### 2.4 Immunohistochemistry staining

After intrinsic peroxidase activity, the articular cartilage sections were blocked with 3% hydrogen peroxide (H_2_O_2_) and then incubated with 1.5% BSA for 1 hour. The tissue sections were covered with the antibodies against SBP2 (12798‐1‐AP, 1:250 dilution), GPX1 (3120‐1, 1:250 dilution), GPX4 (14432‐1‐AP, 1:500 dilution) and SELS (15591‐1‐AP, 1:500 dilution), respectively, which were purchased from Proteintech (Wuhan, China). The samples were incubated at 4°C overnight on a wet box. The sections were rinsed with PBS. Sequentially, they were incubated with biotinylated secondary antibody for 1 hour and DAB reagent (Boster, Wuhan, China) for 5 minute at room temperature. Chromogenic reactions were terminated once claybank regions were observed under the microscope. Rabbit IgG was used as a negative control.

### 2.5 Luciferase reporter assays

Among hundreds of target genes predicted by the TargetScanHuman7.1 (http://www.targetscan.org/) search programs, *hSBP2* was of particular interest (Figure 2A). For luciferase reporter experiments, a 350‐bp DNA segment transcribed from the part of *hSBP2 mRNA 3*′*‐UTR* was amplified by PCR from human cDNA and inserted into the psi‐CHECK‐2 Vector (Promega, Fitchburg, USA) between the Xho I and Not I (Fermentas, Canada) site. The information of mature *miR‐181a‐5p* and stem‐loop miR‐181a is depicted in Tables S1 and S2, respectively.

The primer sequences to generate wild type *hSBP2 3*′*‐UTR* fragment (WT group) are as follows: forward primer: 5′‐atactcgagCAGGGAAAGGGCCCTTT‐3′ and reverse primer: 5′‐acagcggccgcTTTCACCAGAGTCTGAAA‐3′. The mutant *hSBP2 3*′*‐UTR* DNA fragment sized 350 bp was chemically synthesized with binding sequence completely mutated from TGAATGT to ACTCGTA (Figure 2A) and used the same primers with WT group to be amplified. The insert segment was confirmed by sequencing (data not shown).

HEK‐293T cells were seeded in 48‐well plates at 70% density and co‐transfected with 0.3 μg *hSBP2 3*′*‐UTR/hSBP2 3*′*‐UTR‐mutant* plasmid and 50 nmol L^−1^ *mimic‐miR‐181a‐5p* or 200 nmol L^−1^ *inhibitor‐miR‐181a‐5p* (Genepharma, Shanghai, China) in each well, using Lipofectamine 2000 (Invitrogen, Carlsbad, USA). Luciferase assays were performed 48 hour after transfection by using the Dual‐Luciferase^®^ Reporter 1000 Assay System (Promega, Fitchburg, USA) on a multiskan spectrum Luminoskan ascent 392 (Thermo, Waltham, USA).

### 2.6 Cell transfection

Full‐length human *hSBP2* cDNA was amplified and subcloned into the pHBAd‐MCMV‐GFP expression vector. Null vectors (without exogenously inserted DNA fragment) were used as the controls. The pHBAd‐MCMV‐GFP‐SBP2(Ad‐SBP2), a recombinant adenoviruses containing *hSBP2‐CDS* segment, and control (Ad‐GFP) were packaged by Hanbio Biotechnology Inc. (Hanbio, Shanghai, China).

The siRNA of *hSBP2* (*si‐SBP2*) and control siRNA sequences was purchased from Genepharma Biotechnology Inc. (Genepharma, Shanghai, China). Cell transfection was performed according to the manufacturer's instructions. To obtain gene overexpression, the recombinant adenoviruses were used as 300 MOI of final viral titre and added to the cells for incubation. For gene knockdown, the best fit siRNA of *hSBP2*,* hSBP2‐siRNA2527*: GAGCCACACUACAUUGAAAtt, was transduced into the cells according to the manufacturer's instructions.

### 2.7 Total RNA extraction and quantitative PCR analysis

Total RNA was isolated from rats articular cartilage or C28/I2 cell samples using the TRIzol^®^ method (Invitrogen, USA). Two microgram of each total RNA from articular cartilage was timely reversed to cDNA according to the manufacturer's instructions (RevertAid^™^; Fermentas, Canada) and stored at −20°C until used. In addition, miRNA‐cDNA was obtained using One Step PrimeScript^®^ miRNA‐cDNA Synthesis Kit (Takara, Japan). Because the cartilage were limited and contained very less RNA in one sample, it could not satisfy the test of every gene.

The mRNA or miRNA expression was tested by real‐time quantitative PCR (RT‐qPCR), which was performed on iQ5 real‐time PCR detection system (Bio‐Rad, Hercules, CA, USA) with SYBR^®^ Premix Ex Taq^™^ II (TaKaRa, Japan). The relative gene expression was normalized by GAPDH, while let‐7a was used as the internal reference of *miR‐181a‐5p*. The procedure of miRNA‐cDNA qPCR was two‐step amplification:pre‐degeneration 95°C 10 second; PCR amplification: 95°C 5 second, 60°C 20 second, 40 cycles. The information of primers is depicted in Tables 1, 2, 3.

**Table 1:** Information of miRNA‐181a‐5p for real‐time PCR

MicroRNAs	Accession No.	Forward primer
*hsa‐miRNA‐181a*	MIMAT0000858	5′‐CGCAACATTCAACGCTGTC‐3′
*hsa‐let‐7a*	MIMAT0000774	5′‐CGCTGAGGTAGTAGGTTGT‐3′
Reverse primer: 5′‐ GTGCAGGGTCCGAGGT‐3′		

**Table 2:** Information of rat primers for real‐time PCR

Gene	Sequence (5′‐3′)	Product size (bp)	Annealing temperature (°C)
*Sbp2*	Forward: AAGGCACCCTAGCTTCCACT	116	60
	Reverse: GGTTGATGCCTCCAGTGAGT		
*GPx1*	Forward: GGCTCACCCGCTCTTTACCTTC	168	62
	Reverse: CGCACTGGAACACCGTCTGG		
*GPx4*	Forward: AATTCGCAGCCAAGGACATCG	168	62
	Reverse: CCAGGATTCGTAAACCACACTCG		
*SelS*	Forward: TTCCTGCACGTCACAGTGGG	115	60
	Reverse: TTAAAGCCCTCAGTCGCAGG		
*GAPDH*	Forward: CGGCAAGTTCAACGGCACAG	148	60
	Reverse: GAAGACGCCAGTAGACTCCACGAC		

**Table 3:** Information of human primers for real‐time PCR

Gene	Sequence (5′‐3′)	Productsize (bp)	Annealing temperature (°C)
*SBP2*	Forward: CCGCAGATTCAGGGATTACT	92	60
	Reverse: CTTGGAAACGGACCAGTTCT		
*GPx1*	Forward: AAGCTCATCACCTGGTCTCC	124	60
	Reverse: CGATGTCAATGGTCTGGAAG		
*GPx4*	Forward: GCTGTGGAAGTGGATGAAGA	105	60
	Reverse: TGAGGAACTGTGGAGAGACG		
*SelS*	Forward: CACCTATGGCTGGTACATCG	130	60
	Reverse: AACATCAGGTTCCACAGCAG		
*GAPDH*	Forward: CACCCACTCCTCCACCTTTG	110	64
	Reverse: CCACCACCCTGTTGCTGTAG		

### 2.8 Western blotting

Total protein were isolated and analysed by *Western blotting*. Protein samples from rats articular cartilage or C28/I2 cell line (10‐20 μg) were separated on 10% SDS‐PAGE and transferred to PVDF membranes (EMD Millipore, Darmstadt, Germany). After blocked with 3% non‐fat milk in TBST buffer, membrane was incubated with primary antibodies followed by the secondary antibodies conjugated with horseradish peroxidase (HRP), and visualized using the ECL detection system (EMD Millipore, Darmstadt, Germany) on a chemiluminescence imaging system. The antibodies against SBP2 (12798‐1‐AP, 1:500 dilution), GPX1 (3120‐1, 1:2000 dilution), GPX4 (14432‐1‐AP, 1:500 dilution) and SELS (15591‐1‐AP, 1:1000 dilution) were from Epitomics (CA, USA). Antibody against β‐actin (1:2000 dilution), the secondary horseradish peroxidase‐coupled anti‐rabbit (1:5000 dilution) and anti‐mouse (1:5000 dilution) antibodies were purchased from Byotime Biotech (Jiangsu, China).

### 2.9 Statistical analysis

Data were presented as means ± SEM. The statistical significance of pathological data was calculated by Mann‐Whitney U test. Means of two groups were compared using Student's *t* test, and statistical significance was considered as *P* < 0.05 in all tests (*P < 0.05, **P < 0.01and **P < 0.001). All analyses were performed using the software GraphPad Prism 6.0 (GraphPad Software, San Diego, CA, USA).

## 3 RESULTS

### 3.1 Both *miR‐181a‐5p* and SBP2 are selenium‐sensitive in cartilage and chondrocytes

**Figure 1:**
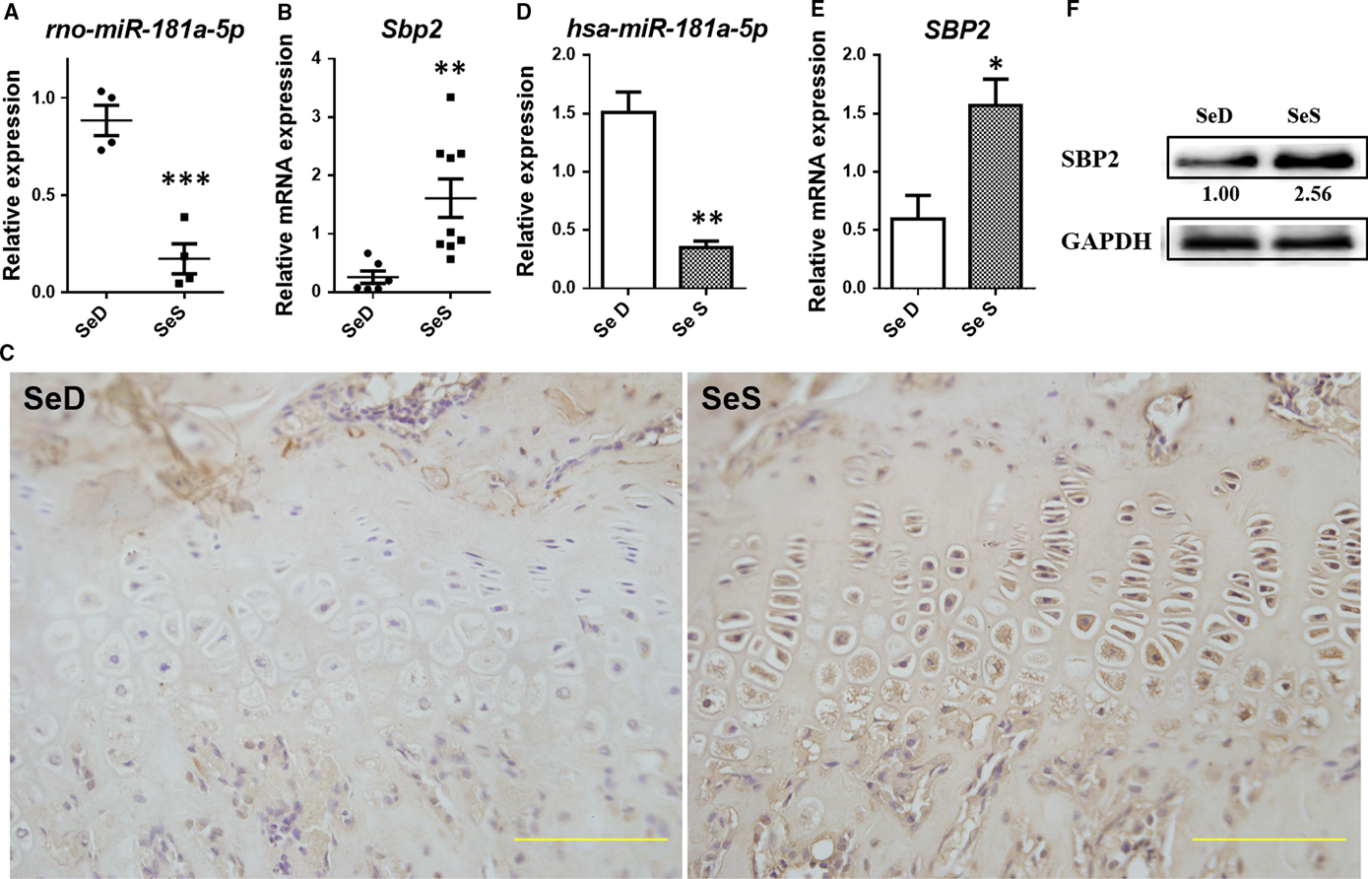
Both *miR‐181a‐5p*and SBP2 are Se‐sensitive. (A) *rno‐miRNA‐181a‐5p* expression in low Se rat cartilage (n = 4, 4). (B) *Sbp2* expression in low Se rat cartilage (n = 6, 9). (C) SBP2 expression in low Se rat cartilage (bar: 50 μm). (D) *hsa‐miRNA‐181a‐5p* expression in C28/I2 chondrocytes under Se deficient or Se replenished condition. (E) *SBP2* expression in C28/I2 chondrocytes under Se deficient or Se replenished condition. (F) SBP2 expression in C28/I2 chondrocytes under Se deficient or Se replenished condition. Data were presented as means ± SEM. *, ** and *** stand for *P* < 0.05, 0.01 and 0.001, respectively, between SeS and SeD groups

Firstly, the expressions of *miR‐181a‐5p*,* Sbp2* and SBP2 were detected in articular cartilage of the low Se diet rat model.^9^ In SeD rat cartilage, *miR‐181a‐5p* showed remarkable up‐regulation as compared to the SeS group (*P* = 0.0006, Figure 1A). Conversely, the expression of SBP2 was significantly reduced in SeD group compared with SeS group, both at mRNA and protein levels (*P* = 0.0066, Figure 1B and C). It seemed to be in line with our expectation that low Se could result in *Sbp2* down‐regulation in cartilage, with *miR‐181a‐5p* up‐regulated. In other words, SBP2 was significantly down‐regulated in SeD groups while up‐regulated in SeS groups regardless of interferring or overexpressing *Sbp2* and SBP2 (Figure 1A‐C).

Further, a seven‐day‐term low medium cell culture was performed as described previously to induce the low Se condition in vitro.^25^ *MiR‐181a‐5p* expressed at significantly elevated levels in SeD group compared with Se supplement cultured C28/I2 cells (*P = *0.0035, Figure 1D), indicating that *miR‐181a‐5p* was Se susceptive in chondrocytes, which was in line with the results obtained from the rat model, presented above. Meanwhile, *SBP2*and SBP2 were significantly down‐regulated, contrarily (*P* = 0.0335, Figure 1E and F). It is evident from the above results that both *miR‐181a‐5p* and SBP2 are selenium‐sensitive and are regulated by Se supplement.

### 3.2 SBP2 is a direct target of *miR‐181a‐5p* in C28/I2 cells

**Figure 2:**
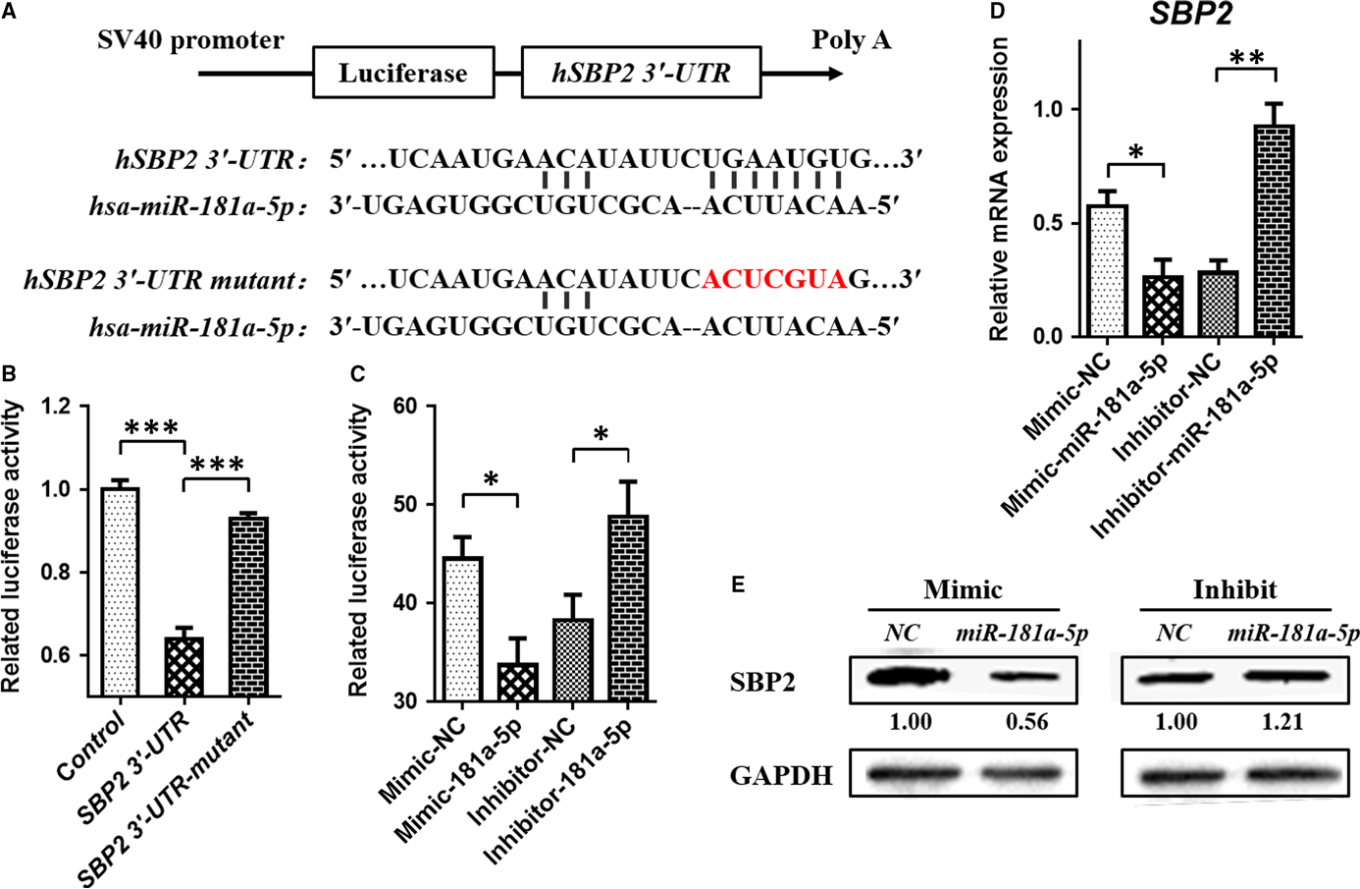
There is a target relationship between *miRNA‐181a‐5p* and *SECISBP2*. (A) A Schematic diagram of hsa‐miRNA‐181a‐5p binding site with SBP2 3’‐UTR. (B) Results of dual‐luciferase reporter gene analysis with wild or mutant SBP2 3’‐UTR. (C) Results of dual‐luciferase reporter gene analysis with mimic or inhibitor transfected in C28/I2 chondrocytes. (D) mRNA expression of SBP2 in C28/I2 cell line transfected with mimic or inhibitor of hsa‐miRNA‐181a‐5p. (E) Protein expression of SBP2 in C28/I2 cell line transfected with mimic or inhibitor of hsa‐miRNA‐181a‐5p. Data were presented as means ±  SEM. *, ** and *** stand for *P* < 0.05, 0.01 and 0.001, respectively

Subsequently dual‐luciferase reporter gene analysis showed that, the relative activity of Rluc/Fluc of *SBP2* WT group was reduced about 37%, compared with the control group, prompting that *hsa‐miR‐181a‐5p* could bind with *SBP2‐3′‐UTR* so as to affect Rluc gene expression. Compared with WT group, the relative activity of Rluc/Fluc of *SBP2* mutant group Rluc/Fluc was increased about 30%. Furthermore, there was no significant difference between mutant group and control group, suggesting that *hsa‐mir‐181a‐5 p* could not bind with *SBP2‐3′‐UTR* because of the mutation in the predicted binding sites (*P* < 0.0001, Figure 2B).

Meanwhile, miR‐24‐3p did not target the *SBP2‐3′‐UTR* in C28/I2 cells although it was predicted. (Figure S1).

Then, *mimic‐miR‐181a‐5p* and *inhibitor‐miR‐181a‐5p* were used to alter the level of *miR‐181a‐5p*, and the relative activity of dual‐luciferase reporter gene and *SBP2* expression were detected. *Mimic‐miR‐181a‐5p* could down‐regulate the dual‐luciferase activity (*P* *=* 0.0147), while the *inhibitor‐miR‐181a‐5p* could up‐regulate it significantly (*P* *=* 0.0440) (Figure 2C), in accord with the changes of SBP2 mRNA (*P* *=* 0.0364, *P* *=* 0.0048, Figure 2D) and protein (Figure 2E) levels. These results suggest that *hsa‐miR‐181a‐5p* can specifically bind with the *SBP2* mRNA 3′‐UTR. In other words, *SBP2* is a direct target gene of *miR‐181a‐5p*.

### 3.3 Both *miR‐181a‐5p* and SBP2 could regulate the biosynthesis of three selenoproteins in chondrocytes

**Figure 3:**
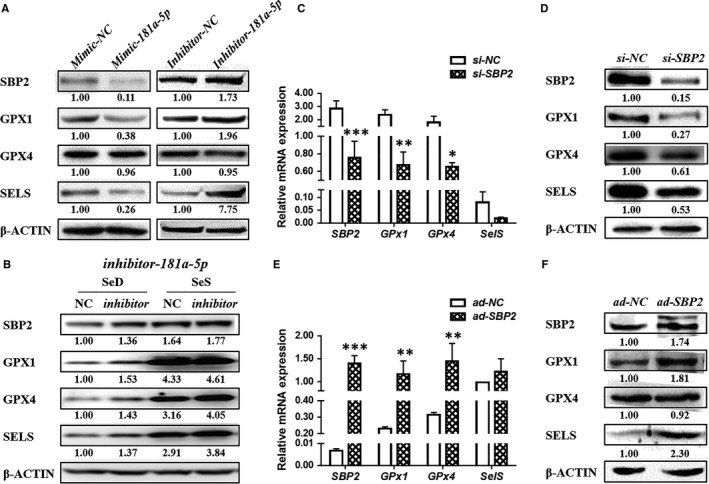
Both *miR‐181a‐5p*and SBP2 regulate the expression of 3 selenoproteins in chondrocytes. (A) Left: The protein expressions of SBP2 and three selenoproteins in C28/I2 cell line transfected with mimic of *hsa‐miRNA‐181a‐5p*. Right: The protein expressions of SBP2 and three selenoproteins in C28/I2 cell line transfected with inhibitor of *hsa‐miRNA‐181a‐5p*.(B) The protein expressions of SBP2 and selenoproteins after *inhibitor‐181a‐5p* infection in Se deficient or Se replenished C28/I2 chondrocytes.(C) The protein expressions of SBP2 and 3 selenoproteins in C28 cells transfected with *siRNA‐SBP2*. (D) The mRNA expressions of SBP2 and 3 selenoproteins in C28 cells transfected with *siRNA‐SBP2*.(E) The protein expressions of SBP2 and 3 selenoproteins in C28 cells infected with Ad‐SBP2 plasmid.(F) The mRNA expressions of SBP2 and 3 selenoproteins in C28 cells infected with Ad‐SBP2 plasmid. Data were presented as means ± SEM. *, ** and *** stand for *P* < 0.05, 0.01 and 0.001, respectively.

The mimic or inhibitor of *miR‐181a‐5p* was used in normal C28/I2 cells to check the expression of SBP2 and the three selected selenoproteins. After *mimic‐miR‐181a‐5p* transfection, Western blotting results showed that SBP2, GPx1 and SELS were significantly down‐regulated, while there was no change in GPX4 levels (Figure 3A left). On the other hand, inhibitor‐miR‐181a‐5p resulted in the up‐regulation of SBP2, GPx1 and SELS proteins levels except the GPX4 (Figure 3A right).

Moreover, after *miR‐181a‐5p* inhibition in Se deficiency and Se supplemented chondrocytes, both the groups showed an elevation in the expression of SBP2. And, the three selenproteins were also increased in the inhibitor groups compared to the respective control groups.(Figure 3B).

To assess the role of SBP2, regulating the selenoproteins in chondrocyte, we detected the expression of *GPx1*,* GPX4* and *SELS* in human C28/I2 cells with knockdown or overexpression of SBP2, at RNA and protein levels, respectively. When SBP2 (*P* *=* 0.0002) level was reduced by specific siRNA, mRNA levels of *Gpx1* (*P* *=* 0.0014) and*GPX4* (*P* *=* 0.0201) were down‐regulated significantly except *SELS* (*P* *=* 0.8900) (Figure 3C); however, at protein level, all the three selenoproteins were significantly down‐regulated (Figure 3D). On the other hand, SBP2 (*P* *=* 0.0002) overexpression in C28/I2 cells could elevate the mRNA expression of both *Gpx1* (*P* *=* 0.0059) and *GPX4* (*P* *=* 0.0014) but could not change *SELS* mRNA levels ( 3E). At the protein level, GPX1 and SELS had the obvious response to the SBP2 overexpression, whereas GPX4 showed no evident changes (Figure 3F). These findings suggest that SBP2 can affect the pivotal selenoproteins expression in chondrocyte.

### 3.4 SBP2 regulates selenoproteins in different Se conditions

In order to further explore the role of SBP2 in cartilage, the knockdown or overexpression of SBP2 was performed in C28/I2 cells by *si‐SBP2* or ad‐SBP2, respectively. Under the Se deficient or Se replenished situations, the chondrocytes were treated with *si‐SBP2* or Ad‐SBP2, respectively, in order to observe the expression of the above three selected selenoproteins.

**Figure 4:**
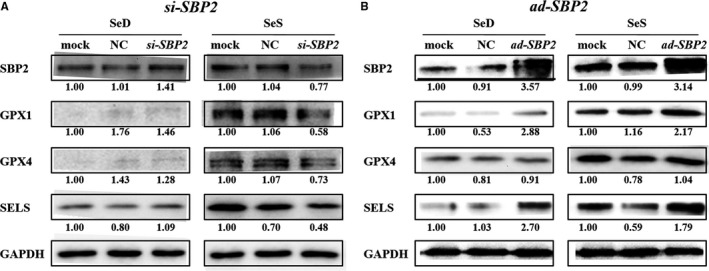
Selenoproteins expression in C28 cells after *si‐RNA SBP2* transfection. (A) The protein expressions of SBP2 and selenoproteins after *si‐SBP2* transfection in Se deficient or Se replenished C28/I2 chondrocytes. (B) The protein expressions of SBP2 and selenoproteins after Ad‐SBP2 plasmid infection in Se deficient or Se replenished C28/I2 chondrocytes

In SeD groups of *si‐SBP2*, the expression of SELS was very low, even GPX1 and GPX4 were difficult to detect, no matter transfected with *si‐SBP2* or not. All concerned proteins could be visibly detected with Se supplement, showing a reduced tendency in the *si‐SBP2* chondrocytes (Figure 4A).

In the SBP2 overexpression chondrocytes, both SeD and SeS groups showed a remarkable elevation in the expression of SBP2. Interestingly, the GPX1, GPX4 and SELS were robustly up‐regulated compared with control group despite selenium deficient conditions. After the selenium was replenished in to the selenium deficient chondrocytes, the selected selenoproteins expression was rescued significantly. On the other hand, GPX4 showed a little response to the SBP2 overexpression in selenium deficient or supplement chondrcocytes (Figure 4B).

### 3.5 The selected selenoproteins are down‐regulated in cartilage of low Se diet rat

**Figure 5:**
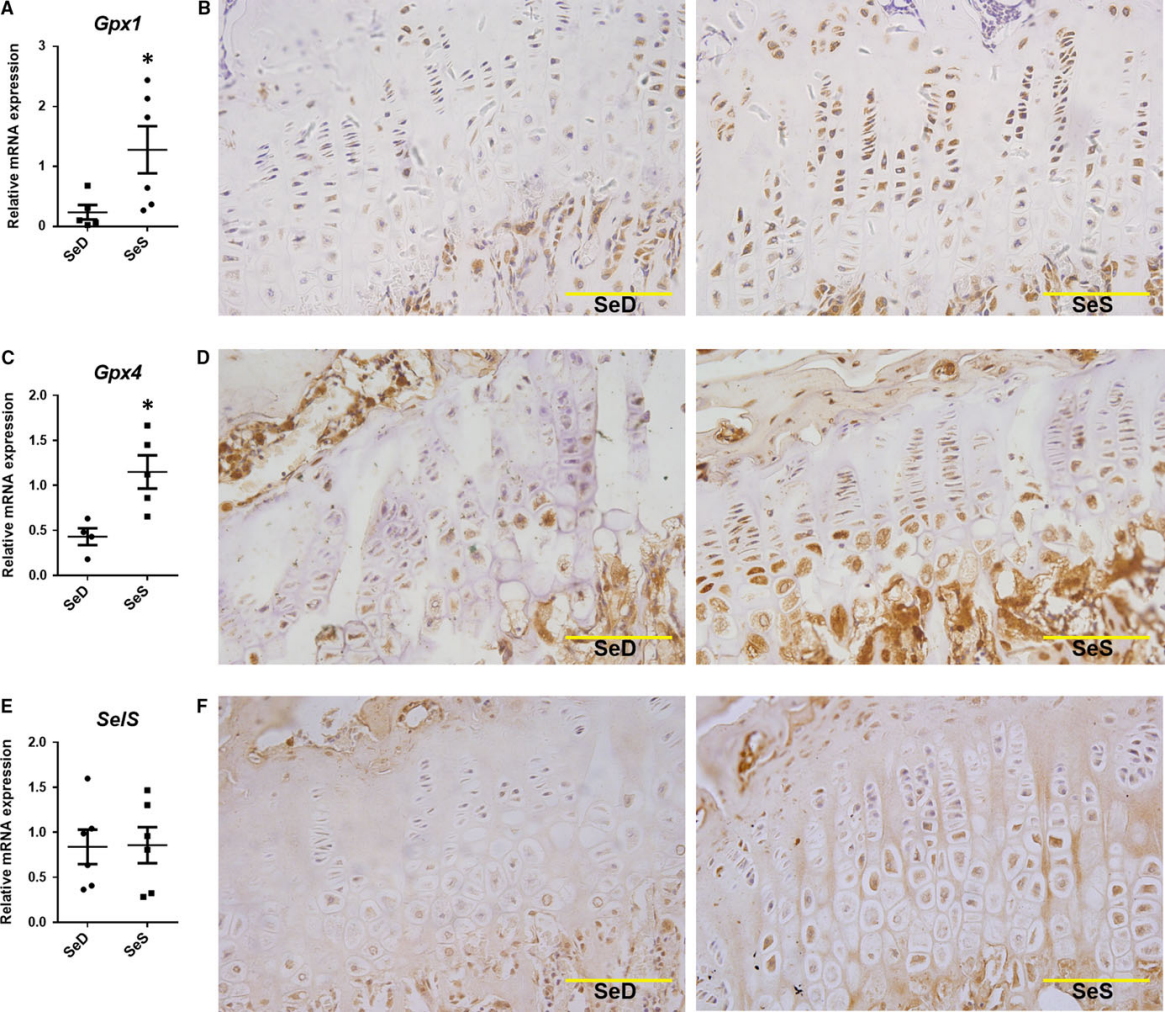
Expression of three selenoproteins in low Se DA rats cartilage model. (A) *GPx1* mRNA expression in low Se rats cartilage(n = 6, 5).(B) IHC results of GPx1 protein expression in low Se rats cartilage(bar: 50 μm).(C) *GPx4* mRNA expression in low Se rats cartilage (n = 4, 5).(D) IHC results of GPx4 protein expression in low Se rats cartilage(bar: 50 μm).(E) *Sel S* mRNA expression in low Se rats cartilage (n = 6, 6).(F) IHC results of Sel S protein expression in low Se rats cartilage(bar: 50 μm). Data were presented as means ± SEM. * stand for *P* < 0.05, respectively, between SeS and SeD groups

In the low Se diet rat model, expression of *GPx1*,* Gpx4* and *SelS* was determined by RT‐qPCR and IHC methods. In the epiphyseal plate of low Se DA rats cartilage (SeD group), mRNA expression of *Gpx1* (*P* *=* 0.0449, Figure 5A) and *Gpx4* (*P* *=* 0.0154, Figure 5C) was significantly higher than the control (SeS group) group, while *SelS* showed no considerable change in the expression at mRNA level (*P* *=* 0.9489, Figure 5E). However, at the protein level, GPX1 (Figure 5B), GPX4 (Figure 5D) and SELS (Figure 5F) were all significantly down‐regulated, as determined by IHC. These results suggested that *GPx1*,* GPX4* and *SelS* participate in the low Se induced cartilage lesions.

## 4 DISCUSSION

To investigate whether *miR‐181a‐5p* is involved in cartilage injury process induced by Se deficiency, we established the Se deficiency and Se sufficiency models using C28/I2 human chondrocytes and inbred DA rats.^9^ The phenotypes of the two generation Se deficiency rats included extracellular matrix (ECM) metabolism, aberration of articular cartilage, epiphyseal dysplasia, delayed skeletal development and the secondary osteoarthritis.^9^, ^27^ These phenotypes match exactly with KBD for its Se‐deficient nutritional status.^10^, ^11^, ^12^ Moreover, the main features of epiphyseal dysplasia are the Collagen2α1(COL2α1) and ACAN metabolic imbalances and chondrocytes apoptosis interrelated to abnormal proliferation and differentiation in the Se deficient diet fed rats,^9^, ^27^ similar to the symptoms of KBD patients.^10^, ^11^, ^12^ Conformably, *miR‐181a‐5p* down‐regulated the ACAN in chondrocytes through the unknown mechanism.^24^ Predictably, *miR‐181a‐5p* was up‐regulated in Se deficiency both in vivo and in vitro. The expression of *miR‐181a‐5p* showed a decline with Se deficiency, suggesting that *miR‐181a‐5p* is susceptive to Se supply in cartilage.

Next, we used mimic and inhibitor sequences of *miR‐181a‐5p* to up‐ or down‐regulate its expression in C28/I2 cells. The *Sbp2*/*SBP2* showed a significant negative correlation with the *miR‐181a‐5p*. Further, we proved that SBP2 is a new, direct target of *miR‐181a‐5p* in chondrocytes. These results establish that Selenium‐sensitive *miR‐181a‐5p* can regulate the expression of three selected selenoproteins *GPx1*,* GPX4* and *SELS* to a variable extent through its target gene *SBP2* in cartilage.

Meanwhile, the three selenoproteins were out‐of‐step regulated by *miR‐181a‐5p* mediated by SBP2, because their SECIS had the different affinity and binding efficiency with SBP2,^6^, ^7^, ^28^, ^29^, ^30^, ^31^ and SBP2 affects the expression of selenoproteins at mRNA levels.^25^, ^28^, ^29^, ^30^ Unexpectedly, GPX4 would not be regulated by *miR‐181a‐5p* and SBP2 after their knockdown and overexpression in C28/I2 cells. Several mechanisms, such as activated NF‐Y and Sp1, were involved in transcriptional and post‐transcriptional regulation of GPX4.^32^, ^33^ Further, its expression is augmented through post‐transcriptional modification by Grsf1 (guanine‐rich sequence‐binding factor 1) which is the *Gpx4* mRNA‐binding protein.^34^ On contrary, the *Gpx4*expression is decreased after Grsf1 knockdown in mice and leads to developmental retardation of the brain which is similar to the Gpx4^+/−^ mice.^34^, ^35^ It is implied that there are multiple modulators and a very complicated regulatory mechanism from Se supplementation to selenoproteins expression.

On the other hand, being the crucial antioxidant enzymes in vivo, both *Gpx1* and *Gpx4* were up‐regulated in cartilage tissue of the second‐generation Se deficient rats, after they were given the Se sufficient diet, and the two selenoproteins were up‐regulated at post‐transcriptional and translational levels.^25^ Perceptibly, it might be necessary for GPX1 and GPX4 to simultaneously participate in chondrogenesis, even though we cannot exclude the possibility that their decreased expression, that might influence the other selenoproteins. Hence, Se status may lead to the better utilization of selenoproteins to maintain cartilage homoeostasis, and it may be partly involved in selenoproteins’ function during cartilage formation and degeneration. Our IHC results supported the idea during this study.

**Figure 6:**
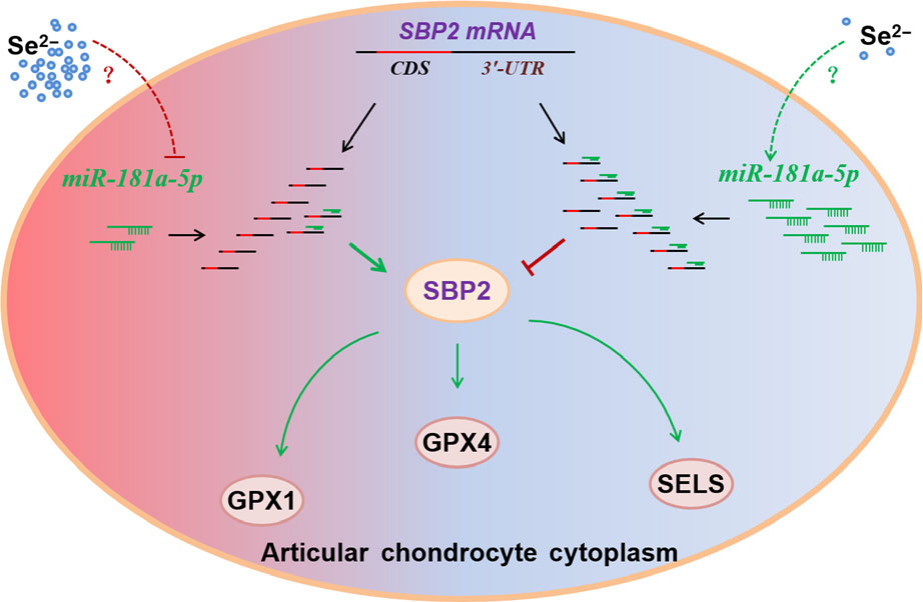
The illustration of possible pathways about selenoproteins regulation by miRNA‐181a‐5p in chondrocytes. Selenium may mediate *miRNA‐181a‐5p* expression may regulate SBP2, resulting in the altered expression of pivotal selenoproteins GPx1, GPx4 and Sel S, which may further play complex roles in cartilage

Finally, we have discovered a novel pathway of Se‐sensitive *miR‐181a‐5p* regulated selenoproteins in cartilage. Selenium can mediate *miR‐181a‐5p* expression, and *miR‐181a‐5p* then regulates SBP2, resulting in the altered expression of pivotal selenoproteins such as GPx1, GPX4 and SELS, which further play complex roles in the cartilage (Figure 6).

In conclusion, the three selenoproteins expression was decreased by Se deficiency which depended on the participation of *miR‐181a‐5p* to down‐regulate SBP2 at post‐transcriptional level. It involves a series of antioxidant and ECM genes, to overcome the ROS‐related stress for the protection of essential physiological functions and to maintain the balance between anabolism and catabolism of the cartilage. Thus, Se deficiency, the risk factor condition, gives rise to epiphyseal dysplasia in DA rats, similar to KBD patients.

Overall, this study provides the first comprehensive evidence that “Selenium → *miR‐181a‐5p *→ SBP2 → selenoproteins” phenomenon exists in cartilage. Therefore, our data suggest that *miR‐181a‐5p*, SBP2 and three pivotal selenoproteins can be used to develop novel diagnostic and therapeutic strategies for cartilage diseases.

## CONFLICT OF INTEREST

The authors have no conflicts of interest to declare.
