# Supplementary figures and images for: Selenium‐sensitive miRNA‐181a‐5p targeting SBP2 regulates selenoproteins expression in cartilage
Source: J Cell Mol Med. 2018 Sep 24;22(12):5888–98. doi: 10.1111/jcmm.13858 (PMC6237606; doi:10.1111/jcmm.13858)

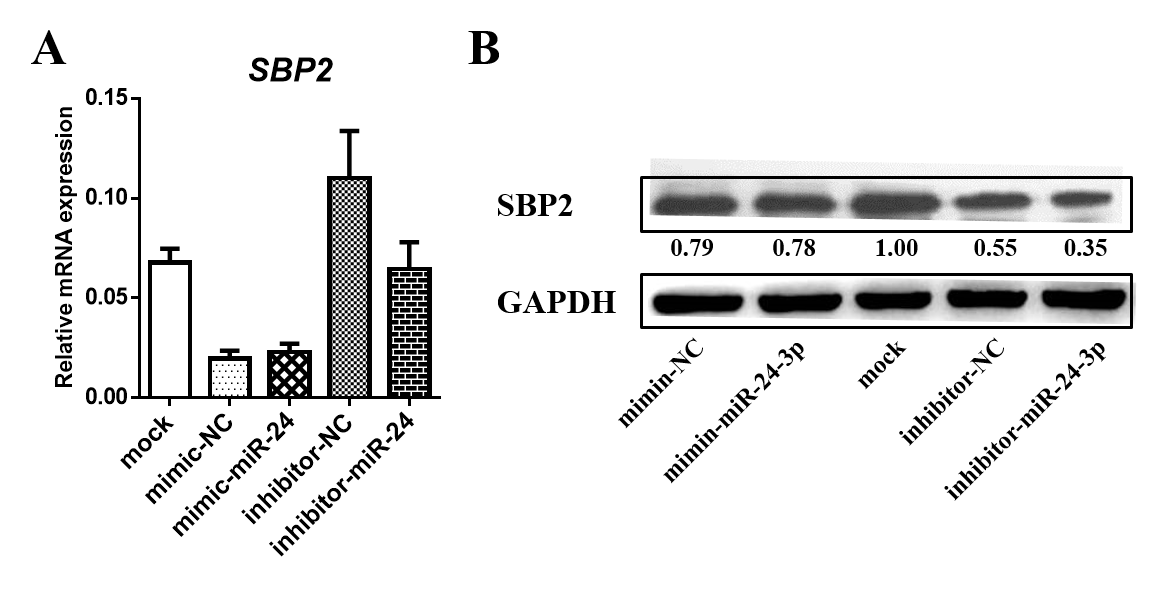

Supplement: Supplementary file 1 [file JCMM-22-5888-s001.tif]

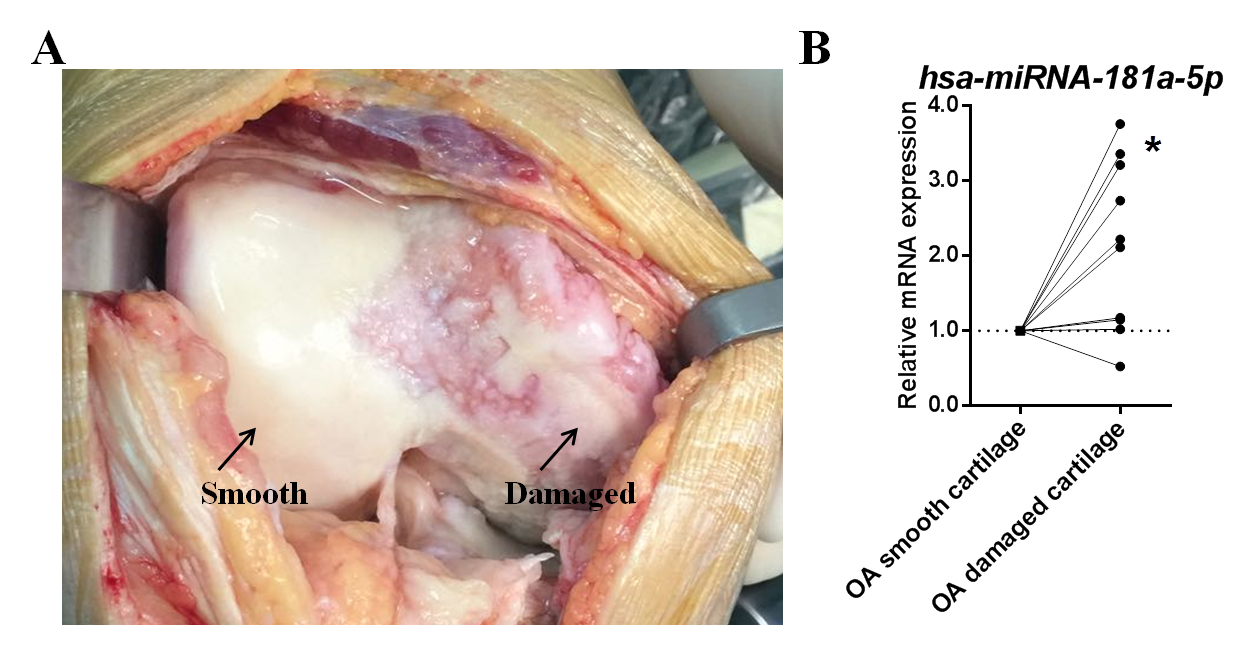

Supplement: Supplementary file 2 [file JCMM-22-5888-s002.png]

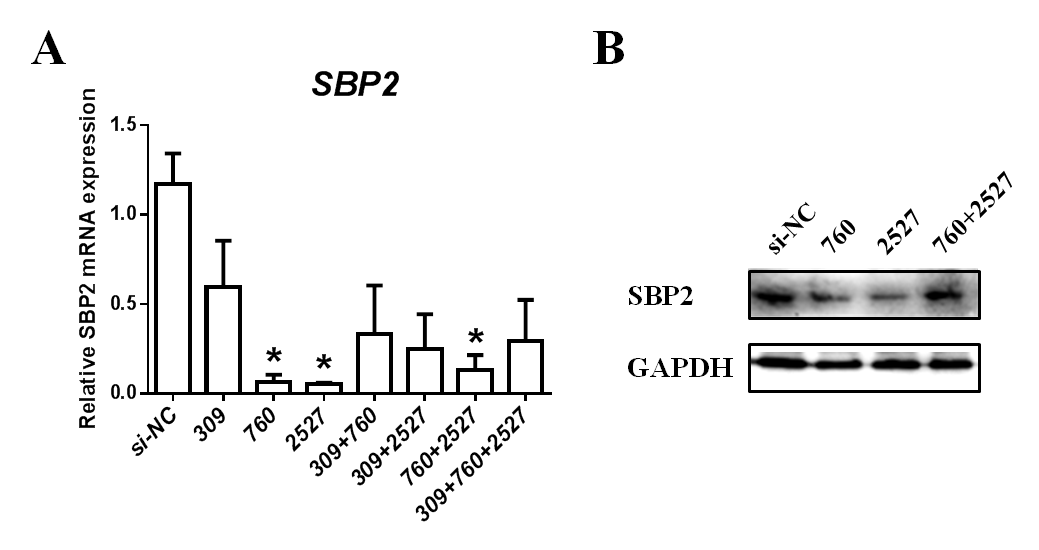

Supplement: Supplementary file 3 [file JCMM-22-5888-s003.png]

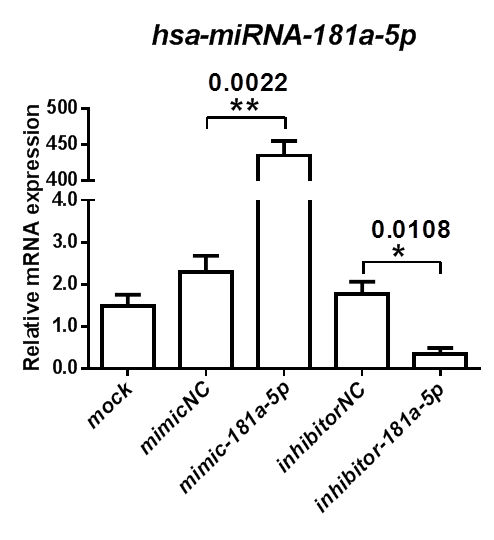

Supplement: Supplementary file 4 [file JCMM-22-5888-s004.png]

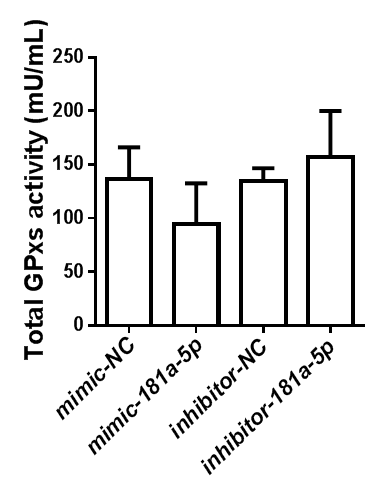

Supplement: Supplementary file 5 [file JCMM-22-5888-s005.png]
