# Supplementary material for: Selenium‐sensitive miRNA‐181a‐5p targeting SBP2 regulates selenoproteins expression in cartilage
Source: J Cell Mol Med. 2018 Sep 24;22(12):5888–98. doi: 10.1111/jcmm.13858 (PMC6237606; doi:10.1111/jcmm.13858)
Supplement: Supplementary file 6 [file JCMM-22-5888-s006.docx]

| Supplementary Tables 1 Information of Mature *miR-181a-5p* | | |
| --- | --- | --- |
| **ID** | **Accession** | **Mature sequence** |
| *hsa-**miR-181a-5p* | MIMAT0000256 | AACAUUCAACGCUGUCGGUGAGU |
| *rno-miR-181a-5p* | MIMAT0000858 |  |
| *mmu-miR-181a-5p* | MIMAT0000210 |  |

| Supplementary Tables 2 Information of Stem-loop *miR-181a* | | | |
| --- | --- | --- | --- |
| **ID** | **Accession** | **Location** | **Stem-loop sequence** |
| *hsa-mir-181a-1*  *(hsa-mir-213)* | MI0000289 | 1q32.1 | UGAGUUUUGAGGUUGCUUCAGUGAACAUUCAACGCUGUCGGUGAGUUUGGAAUUAAAAUCAAAACCAUCGACCGUUGAUUGUACCCUAUGGCUAACCAUCAUCUACUCCA |
| *hsa-mir-181a-2* | MI0000269 | 9q33.3 | AGAAGGGCUAUCAGGCCAGCCUUCAGAGGACUCCAAGGAACAUUCAACGCUGUCGGUGAGUUUGGGAUUUGAAAAAACCACUGACCGUUGACUGUACCUUGGGGUCCUUA |
| *rno-mir-181a-1* | MI0000953 | 13q13 | AGGUUGCUUCAGUGAACAUUCAACGCUGUCGGUGAGUUUGGAAUUCAAAUAAAAACCAUCGACCGUUGAUUGUACCCUAUAGCUAACCAUUAUCUACUCC |
| *rno-mir-181a-2* | MI0000925 | 3q12 | AGAUGGGCAACCAAGGCAGCCUUAAGAGGACUCCAUGGAACAUUCAACGCUGUCGGUGAGUUUGGGAUUCAAAAACAAAAAAAACCACCAACCGUUGACUGUACCUUGGGAUUCUUA |
