# Supplementary material for: Selenium‐sensitive miRNA‐181a‐5p targeting SBP2 regulates selenoproteins expression in cartilage
Source: J Cell Mol Med. 2018 Sep 24;22(12):5888–98. doi: 10.1111/jcmm.13858 (PMC6237606; doi:10.1111/jcmm.13858)
Supplement: Supplementary file 7 [file JCMM-22-5888-s007.docx]

**
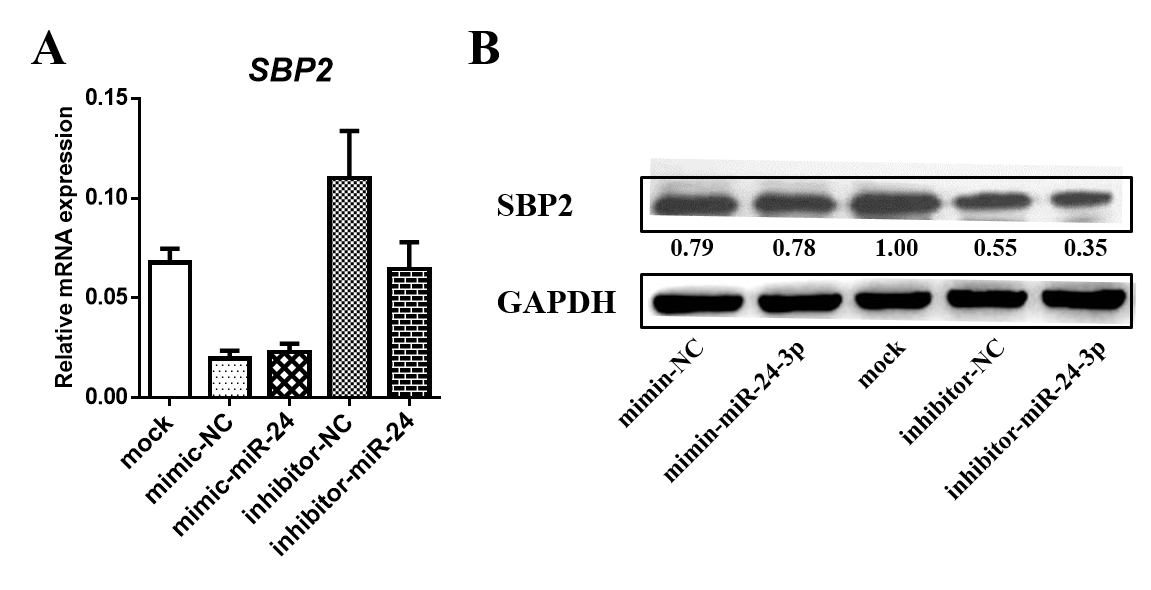
**

**Figure S. There is not a target relationship between *miRNA-24* and *SECISBP2*.**

(A) mRNA expression of SBP2 in C28/I2 cell line transfected with mimic or inhibitor of hsa-miRNA-24. (B) Protein expression of SBP2 in C28/I2 cell line transfected with mimic or inhibitor of hsa-miRNA-24.

Data were presented as means ± SEM. * stand for *P*<0.05.
